# Clinical value of long noncoding RNA ZEB1 anti-sense1 in cancer patients: A meta-analysis

**DOI:** 10.1097/MD.0000000000021307

**Published:** 2020-07-31

**Authors:** Sixiang Cheng, Shengyu Guo, Hairong He, Atipatsa Chiwanda Kaminga, Huilan Xu

**Affiliations:** a Department of Social Medicine and Health Management; b College of Data Science and Information Engineering, Guizhou Minzu University, Guiyang, Guizhou Province; c School of Computer and Communication Engineering, Changsha University of Science and Technology; d Department of Mathematics and Statistics, Mzuzu University, Luwinga, Mzuzu, Malawi; e Department of Epidemiology and Health Statistics, Xiangya School of Public Health, Central South University, Changsha, Hunan, China.

**Keywords:** cancer, clinical value, meta-analysis, ZEB1 anti-sense1

## Abstract

**Background:**

The high expression of long noncoding RNA ZEB1 anti-sense1 (ZEB1-AS1) has been reported in several types of cancer. However, most studies investigating this phenomenon were either case reports or used small patient samples. The objective of this meta-analysis was to clarify the potential clinical values of ZEB1-AS1 in various cancers.

**Materials and methods:**

The PubMed-MEDLINE, Web of Science, and EMBASE databases were searched, using systematic search terms, to find relevant research reports on this subject. The combined hazards ratios (HRs) and corresponding 95% confidence intervals (CIs) were calculated to explore the association between ZEB1-AS1 expression and overall survival (OS). The combined odd ratios (ORs) were calculated to evaluate the association between ZEB1-AS1 expression and pathological parameters. Data analysis was conducted in R software version 3.4.2. and Stata version 12.0 (College Station, TX: Stata Corp LP).

**Results:**

Ten studies including 963 cancer patients were selected as suitable for this study. The pooled hazards ratio (HR) indicated that high ZEB1-AS1 expression was strongly associated with poor OS (pooled HR = 2.26, 95% CI: 1.80–2.85, *P* < .0001) in the Chinese cancer patients. Also, a high expression of ZEB1-AS1 was related to metastasis (pooled HR = 3.38, 95% CI: 1.91–6.00, *P* < .0001), and advanced tumor stage (pooled HR = 0.48, 95% CI: 0.29–0.81, *P* = .005). The up-regulation of ZEB1-AS1 was not significantly associated with histological differentiation (*P* = .39), sex (*P* = .001), and age (*P* = .372) of cancer patients.

**Conclusion:**

The high expression of ZEB1-AS1 significantly predicted poor OS, poor metastasis, and high tumor stage in cancer patients, demonstrating that high ZEB1-AS1 expression may serve as a biomarker of poor prognosis in the Chinese cancer patients.

## Abstract 2

Supplemental Digital Content is available in the text

## 1 Introduction

Cancer, one of the leading causes of death worldwide, is a major public health problem.^[1]^ In China, the number of cancer patients has been increasing in recent years, with an estimated 4,292,000 new cancer cases, and 2,814,000 deaths in 2015.^[2]^ Previous research findings have revealed that crucial to cure cancer is early diagnosis.^[3]^ However, cancer still cannot be diagnosed earlier enough in most cases, although great advances have been made in clinical treatment and management of the disease. One of the reasons affecting tumor therapy and poor prognosis was lack of effective early diagnostic biomarkers.^[4]^ Therefore, new potential biomarkers to guide clinical prevention, treatment, and prognosis of cancer are in need.

Accumulating evidence indicated that, among the key biological factors that are involved in the development and progression of cancer, long noncoding RNAs (lncRNAs) played important roles in tumorigenesis and neoplastic malignant process.^[5–7]^ Besides, LncRNA ZEB1 anti-sense1 (ZEB1-AS1) is located in physical contiguity with ZEB1-AS1, and previous reports indicated that ZEB1-AS1 played critical roles in the progression of different cancers, such as hepatocellular carcinoma,^[8]^ gastric cancer,^[9,10]^ colorectal cancer,^[11,12]^ esophageal carcinoma,^[13]^ glioma,^[14]^ non-small-cell lung cancer, melanoma,^[15,16]^ bladder cancer,^[17,18]^ prostate cancer,^[19]^ and osteosarcoma.^[20]^ Nevertheless, most of the related previous studies have indicated that the downregulation of ZEB1-AS1 was strongly corrected with poor overall survival (OS) and other clinicopathologic characteristics, such as lymph node metastasis, TNM stage, and histological type. However, most studies investigating ZEB1-AS1 in cancer were either case reports or used small patient samples. To date, no meta-analysis has been performed to examine the relationship between ZEB1-AS1 expression and the relevant clinical outcomes.

Therefore, the objective of this study was to analyze all previously published data, based on the robust evidence of the expression and impact of ZEB1-AS1 in tumorigenesis and progression of cancer, to evaluate the clinical value of ZEB1-AS1 in cancer patients.

## 2 Materials and methods

### 2.1 Ethics committee approval

This project was not applicable to or necessary for institutional review board approval due to the fact that it used publicly accessible information, and data of published papers, which was already consented and approved by participants and other ethics review boards, respectively.

### 2.2 Search strategy

Two authors (SXC and GSY) independently conducted the systematic search in the electronic databases of PubMed-MEDLINE, Web of Science, and EMBASE for relevant articles that investigated ZEB1-AS1 as a prognostic biomarker of cancer patients. The search was performed according to the standard guidelines of meta-analysis.^[21,22]^ The latest search was updated on January 20, 2019. Both Text Word[tw] and MeSH strategy were performed with the terms, “ZEB1-AS1” or “LncRNA ZEB1 anti-sense1” or “lncRNA ZEB1-AS1”; “cancer” or “carcinoma” or “tumor” or “tumor” or “neoplasm”; “prognostic” or “prognosis,” or “outcome” or “survival.” These terms were adjusted according to the requirements of the search strategy for a particular database.

### 2.3 Study selection

Two researchers independently evaluated the retrieved information for eligibility for this study and extracted the data. Inclusion criteria were as the following: studies were on the relationship between ZEB1-AS1 and cancer (carcinoma); studies grouped cancer patients based on the level of ZEB1-AS1 expression; studies provided data of odds ratios (ORs) or hazards ratios (HRs) and corresponding 95% confidence intervals (CIs), or these could be derived or obtained from the respective authors upon request; and studies were written in English.

Information from the following studies was excluded: case reports, reviews, editorials, expert opinions, letters, conference abstracts, and animal trials; all studies without usable or sufficient data; and studies not written in English.

### 2.4 Data extraction

Two authors (SXC and SYG) independently extracted relevant data from the eligible studies using a standard data extraction form. Any disagreements between them were resolved through consensus involving a third reviewer (HLX). Therefore, the following data were extracted from each eligible study: surname of first author; study country; year of publication; cancer type; total cases; numbers of patients in the high and low ZEB1-AS1 expression groups; assessment methods; number of patients with Lymph node metastasis (LNM) in each group; and HRs and corresponding 95% CIs. We asked the corresponding authors for relative data if their studies had not provided the data directly, or we derived it from the Kaplan–Meier curve. Egger Digitizer 4.1 version (in China, Beijing) was used to obtain the coordinate values of points on the Kaplan–Meier curves, and a spreadsheet in Microsoft Excel 2010 that implemented all calculations of Tierney methods was used to estimate the HR and corresponding 95% CI.^[20]^

The methodological quality of all included studies was independently assessed using the modified version of the Newcastle-Ottawa Scale (NOS),^[23]^ regarding the up-regulated lncRNA ZEB1-AS1 expression for OS, and reference gene of ZEB1-AS1. Moreover, the study quality could also be evaluated based on the NOS score, which ranges between 0 and 9. In this regard, a study with a score of ≥7 would be deemed to have high quality. The detailed process can be found in Table S1 (see Table S1 in Supplemental Content, which demonstrates the quality scores of included studies on RNA ZEB1-AS1 and OS).

### 2.5 Statistical analysis

All the statistical analyses in this study were performed using R software version 3.4.2. in the “meta” package^[24]^; and Stata version 12.0 (College Station, TX: Stata Corp LP, College Station, TX). The heterogeneity between studies was quantified by the *I*^2^ statistic and evaluated by the *Q* test. An *I*^2^ > 50%, and *P* < .05 was considered to indicate existence of significant heterogeneity. Therefore, a random effects model was used to summarize the effect sizes, HRs, or ORs,^[20]^ when significant heterogeneity was observed; otherwise a fixed effects model was used. In the event that some important data were not reported in an eligible study, we contacted the corresponding author to supply us with the data directly, or we derived it from the Kaplan–Meier curve. The potential publication bias was assessed by Begg funnel plot and Eegg test. The effect of a single article on the heterogeneity and overall risk estimated was assessed by rerunning the analysis each time 1 article is removed successively. A 2-tailed value of *P* < .05 was considered statistically significant.

## 3 Results

### 3.1 Study identification and selection

**Figure 1:**
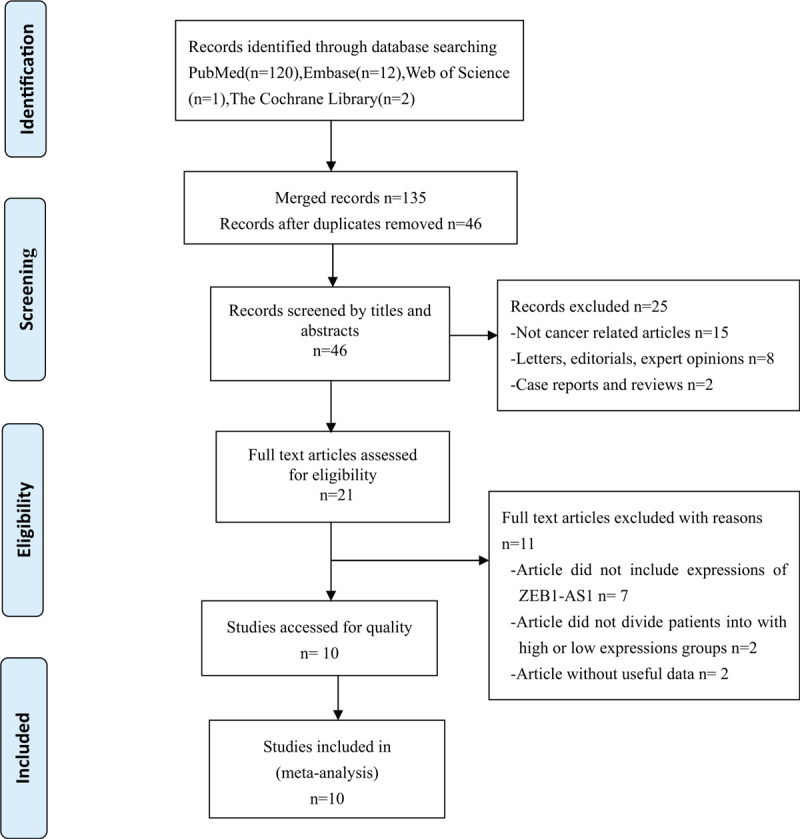
Flowchart presenting the steps of the literature search and selection.

A total of 135 articles were identified through a preliminary database search. After the initial screening of abstracts and titles, 89 duplicates were excluded using Endnote X8 (Thomson Reuters, MI). An assessment of the 46 articles left identified 21 articles for further review. After reading these documents carefully, 11 articles were excluded (3 articles did not include expressions of ZEB1-AS1; 5 articles did not divide patients into high and low expressions groups; and 3 articles had insufficient data). Finally, 10 eligible studies involving a total of 963 patients were included in the meta-analysis.^[8–13]^ The detailed screening process is shown in Fig. 1.

### 3.2 Study characteristics

The characteristics of the 10 eligible studies are summarized in Table 1. All the 10 eligible studies were conducted in China and published between February 2014 and January 2019. The respective sample sizes ranged from 50 to 124 patients. Thus, 10 studies had addressed 9 different types of cancer: 2 focused on gastric cancer (GC),^[9,10]^ 2 on colorectal cancer,^[11–12]^ 1 on esophageal squamous (ESC),^[13]^ 1 on bladder cancer (BC) and prostate cancer,^[15,16]^ 1 on hepatocellular carcinoma (HCC),^[8]^ 1 on Glioma (GLO),^[9]^ and 1 on Osteosarcoma (OSC).^[17]^ The ZEB1-AS1 expression level was measured in all cancerous specimens. Furthermore, all diagnoses of LNM and tumor stage were based on pathology. The NOS scores for all included studies were ≥7, which indicates that the methodological quality of included studies was medium or high (Table 1).

**Table 1:**
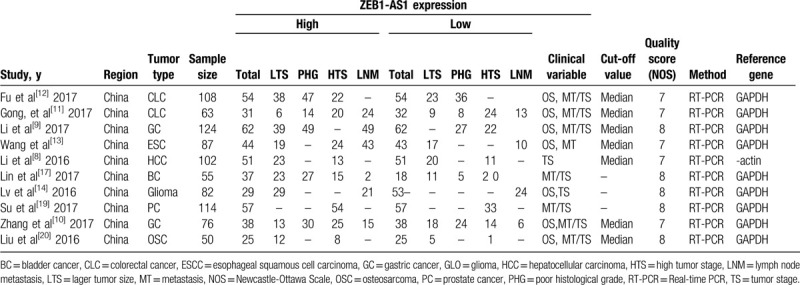
The basic information and data of all included studies in the meta-analysis.

### 3.3 Association between ZEB1-AS1 expression and OS

**Figure 2:**
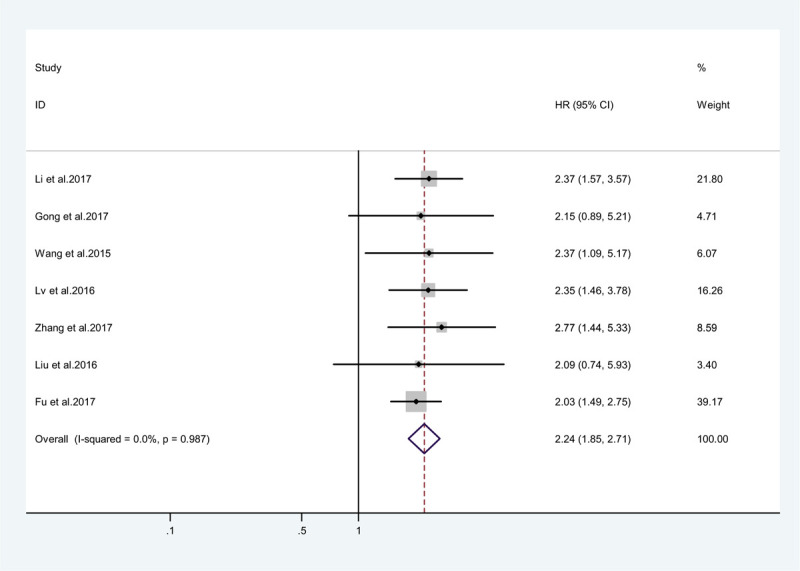
Forest plot showing association between OS and elevated ZEB1-AS1 expression in the different type of cancers. OS = overall survival.

A total of 7 studies with 642 patients had reported the relationship between OS and ZEB1-AS1. Therefore, a cumulative meta-analysis was performed to assess the association between ZEB1-AS1 expression and OS among these patients. The fixed effects model was used due to non-significant heterogeneity (*I*^2^ = 0.000, *P* = .987). According to the statistical analyses, a significant correlation was observed between ZEB1-AS1 and poor OS (pooled HR = 2.269, 95% CI: 1.805–2.853, *P* < .0001; Fig. 2). This result suggested that ZEB1-AS1 could serve as an independent risk factor for OS among cancer patients; besides, high level lncRNA ZEB1-AS1 expression was associated with poor OS.

### 3.4 Association between ZEB1-AS1 expression and metastasis

**Figure 3:**
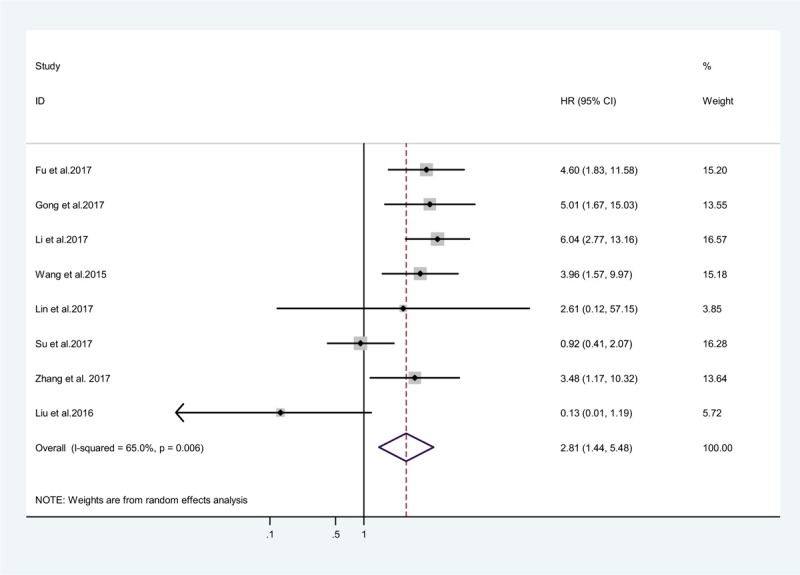
Pooled analysis for the association between ZEB1-AS1 expression and metastasis.

A total of 677 patients from 8 studies were included to detect the relationship between the expression level of ZEB1-AS1 and metastasis. Therefore, the random effects model was used due to significant heterogeneity among the preceding studies (*I*^*2*^ = 65%, *P* = .040). The results showed that there was a significant association between metastasis and ZEB1-AS1 expression (pooled HR = 3.38, 95% CI: 1.91–6.00, *P* < .0001; Fig. 3).

### 3.5 Association between ZEB1-AS1 expression and tumor stage

**Figure 4:**
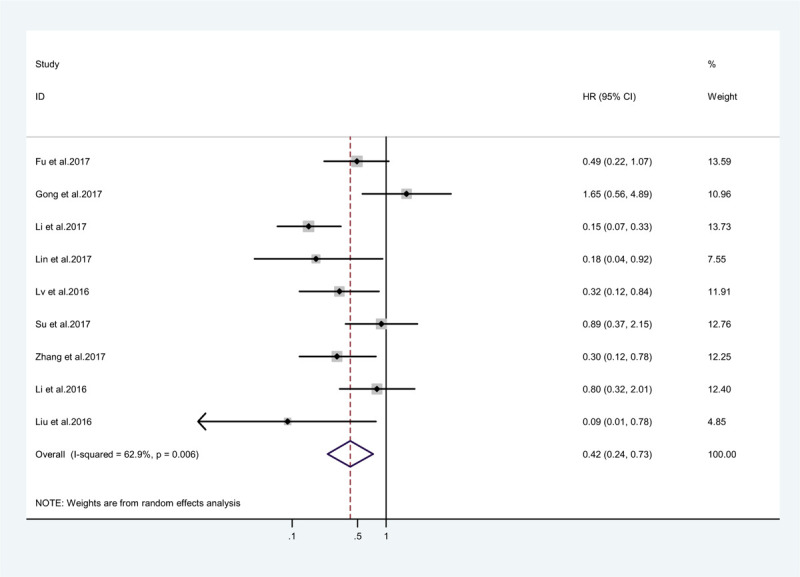
Pooled analysis for the association between ZEB1-AS1 expression and tumor stage.

Among the studies included in the meta-analysis, 9 studies, comprising 876 patients, evaluated the correlation between ZEB1-AS1 expression and tumor stage. A random effects model was used to estimate the correlation because significant heterogeneity among these studies existed (*I*^2^ = 63%, *P* < .010). Therefore, the results revealed that high expression of ZEB1-AS1 was more susceptible to high tumor stage than low expression of ZEB1-AS1 (pooled HR = 0.48, 95% CI: 0.29 to –0.81, *P* = .005; Fig. 4).

### 3.6 Association between ZEB1-AS1 expression and histological differentiation

The role of ZEB1-AS1 expression in tumor histological differentiation was evaluated using a cumulative meta-analysis of 5 eligible studies that reported relevant information for 426 patients. Using a random effects model the results indicated that there was no obvious association between histological differentiation and ZEB1-AS1 expression (pooled HR = 0.66, 95% CI: 0.26–1.69, *P* = .390). A random effects model was used due to significant heterogeneity among the studies (*I*^2^ = 78%, *P* < .010).

### 3.7 Association between ZEB1-AS1 expression and sex/age

Results of the meta-analysis on the association of ZEB1-AS1 expression with sex and age showed that there was no significant association between ZEB1-AS1 expression and sex (pooled HR = 1.244, 95% CI: 0. 981 to –1.579, *P* = .001; fixed effects model) or age (pooled HR = 1.020, 95% CI: 0.802–1.297, *P* = .372; fixed effects model). In this meta-analysis, we found no relationship between the ZEB1-AS1 expression and sex or age in cancer patients, possibly because the included studies applied different detection methods. Studies with larger sample sizes, conducted in other regions and countries other than China, should be carried out in the future.

### 3.8 Subgroup analysis

**Figure 5:**
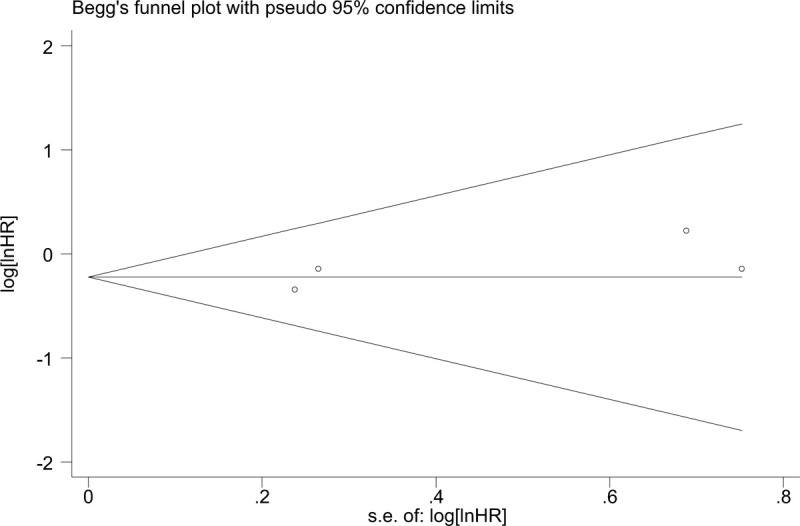
Forest plot of subgroup analysis for OS of patients with cancer: subgroup analysis by tumor type (A), sample size (B), Newcastle-Ottawa Scale, NOS score (C), and HR statistic method (D). OS = overall survival.

Given significant heterogeneity, its source was explored by performing subgroup analyses. Thus, subgroup meta-analyses on the relationship between ZEB1-AS1 expression and OS were performed with regard to the following subgroups: tumor type (digestive system vs non-digestive system); sample size (<100 vs ≥100); NOS scores (7 vs 8); and HR data extraction method (derived from Kaplan–Meier curve vs directly obtained from the paper). The details information were summary in Table 2 and Fig. 5. Therefore, in the subgroup analysis, we found that ZEB1-AS1 expression could serve as the independent factor for predicting OS among cancer patients (HR = 1.99, 95% CI: 1.63, 2.43); besides, high ZEB1-AS1 expression showed correlation with short OS.

**Table 2 Subgroup analysis of overall survival by tumor type, sample size, NOS score,:**
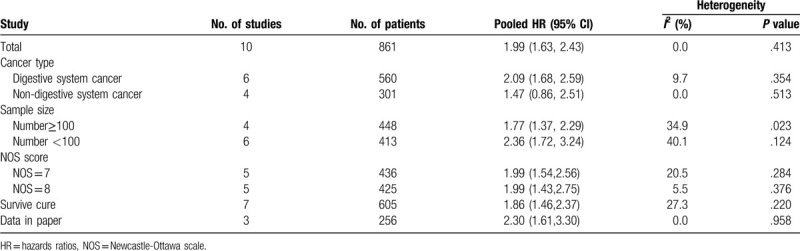
and HR statistic method heterogeneity.

### 3.9 Sensitivity analysis

**Figure 6:**
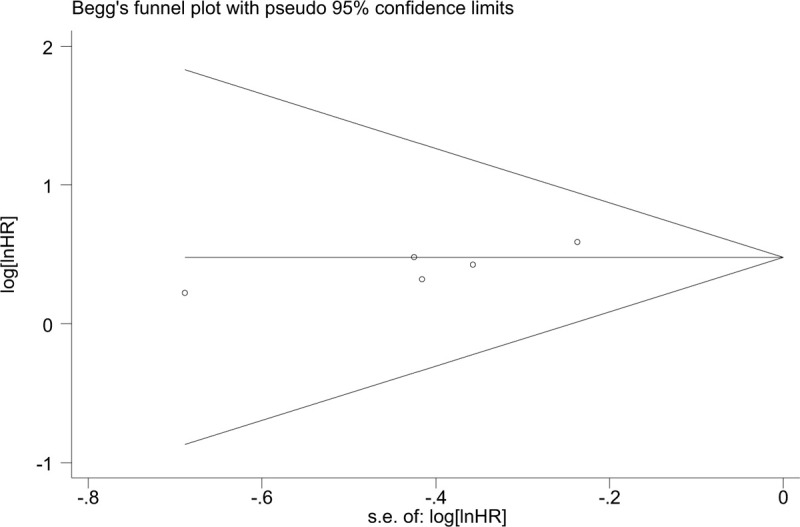
(A) Forest plot and sensitivity analysis (B) showing the association between OS and ZEB1-AS1 expression in cancer patients. OS = overall survival.

To explore the source of statistical heterogeneity of the included studies, sensitivity analyses were conducted. It was found that a study was responsible for most of the heterogeneity.^[13]^ After excluding this study, heterogeneity was much lower among the remaining studies (*I*^2^ = 0.0%, *P* = .633), and the pooled HR was 0.71 (95% CI: 0.59, 0.84). Figure 6 indicates the stable and trustworthy nature of the results of this study.

### 3.10 Publication bias

**Figure 7:**
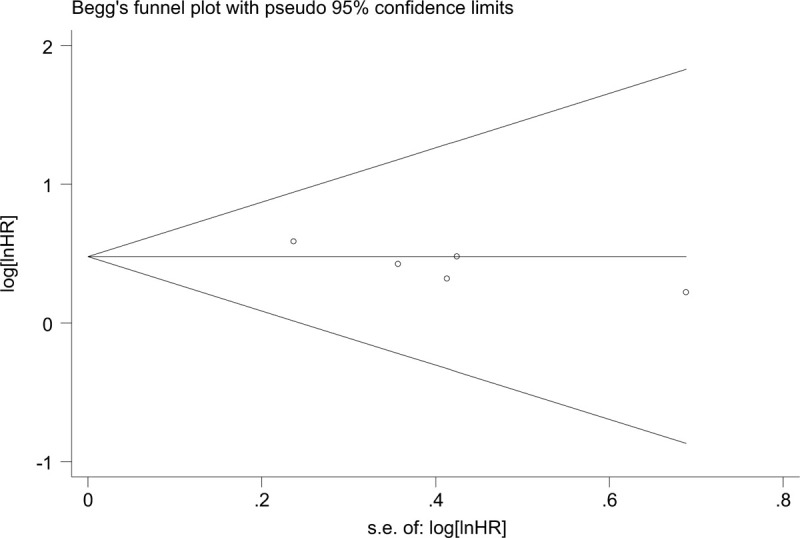
Funnel plot analysis to determine publication bias for the independent role of ZEB1-AS1 and OS. OS = overall survival.

Begg funnel plot was constructed to evaluate publication bias. The results indicated that no significant publication bias existed for the overall survival (Begg test: *P* = .340; Fig. 7), metastasis (Begg test: *P* = .420; Fig. 8 ), and tumor stage (Begg rank correlation test: *P* = .754, Egger test *P* = .367; Fig. 9). The funnel plot did not show any substantial asymmetry Fig. 6A, B.

**Figure 8:**
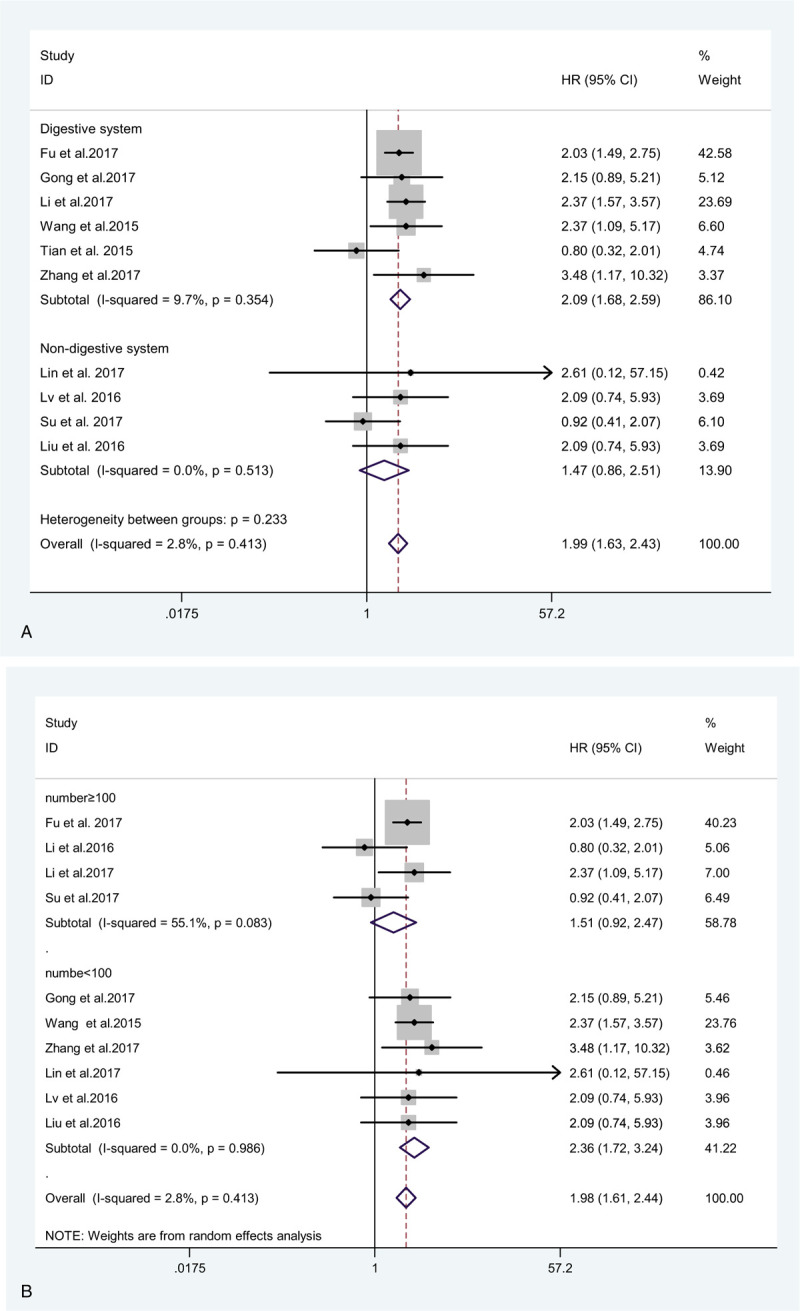
Funnel plot analysis to determine publication bias for the independent role of ZEB1-AS1 and metastasis.

**Figure 8 (Continued):**
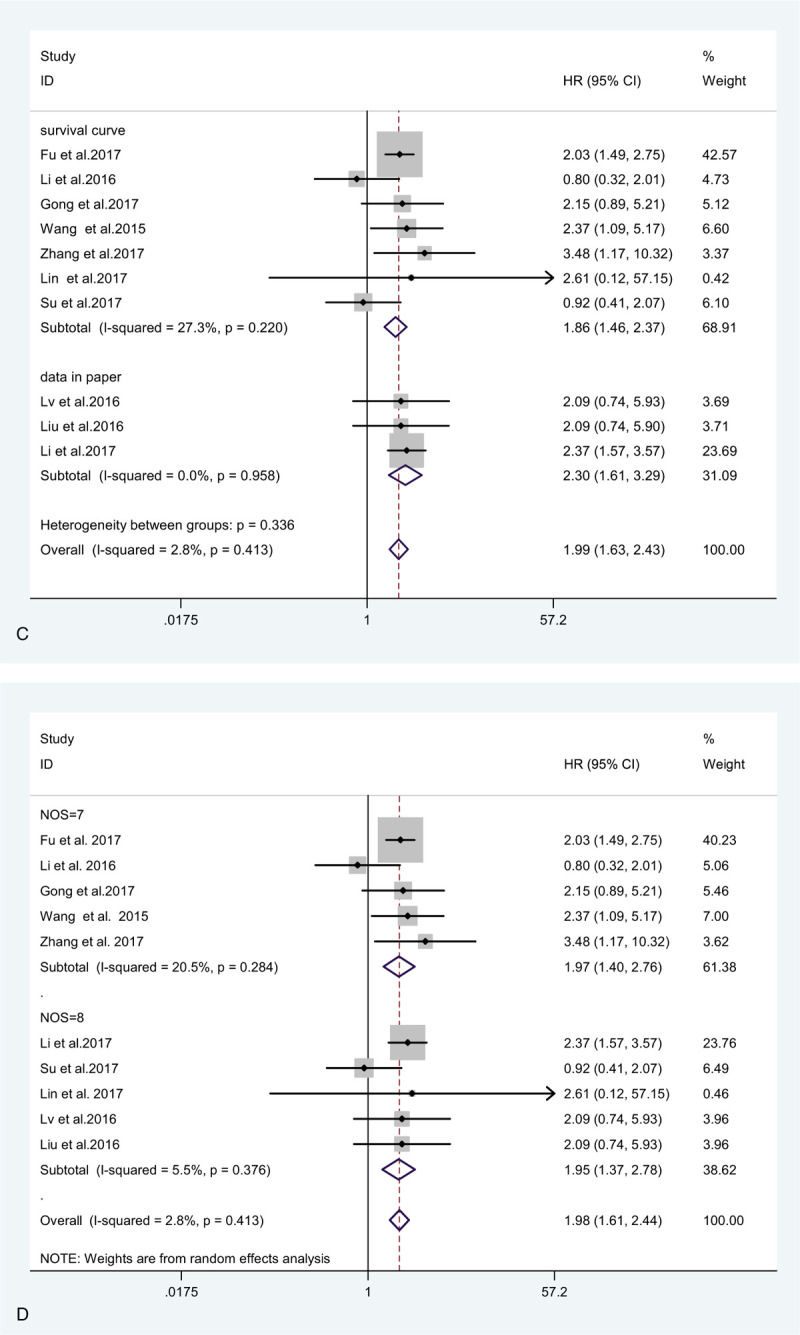
Funnel plot analysis to determine publication bias for the independent role of ZEB1-AS1 and metastasis.

**Figure 9:**
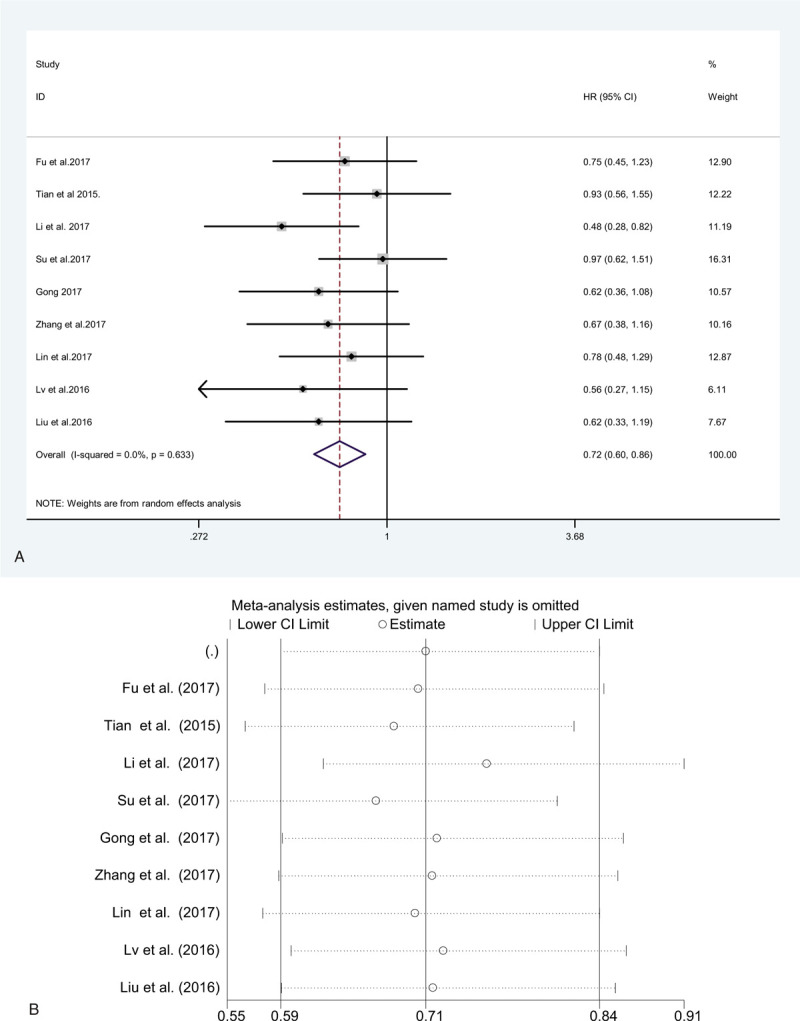
Funnel plot analysis to determine publication bias for the independent role of ZEB1-AS1 and tumor stage.

## 4 Discussion

The average burden of disease due to cancer in China remains increasing in recent years. Despite advancements in clinical treatment of cancer, in terms of reducing pain or prolonging life, early diagnosis is still hard for most cancers, though it is the most effective way to lessen the disease burden.^[25]^ Therefore, new early diagnostic, progressive, and prognostic markers for cancer are urgently required. On this account, it is of great significance to search for novel molecular markers for an accurate prediction of tumor metastasis.

Long noncoding RNAs (lncRNAs) are a class of transcripts longer than 200 nucleotides in length with no protein-coding ability.^[26]^ As newly discovered class of non-coding genes, lncRNAs have been demonstrated to be involved in regulation gene expression, chromatin remodeling, transcription, post-transcriptional RNA processing, and cancer progression.^[27]^ Therefore, lncRNAs could offer a number of advantages as diagnostic and prognostic markers and, also, as novel specific therapeutic targets.

lncZEB1-AS1 has been suggested to be overexpressed and correlated with unfavorable prognosis in many cancers.^[8–11,12]^ Li et al,^[8]^ suggested that gastric carcinoma patients with high expression of ZEB1-AS1 had an elevated risk of the incidence of GC. Additionally, Li et al^[9]^ found that ZEB1-AS1 has been up-regulated in osteosarcoma, which had predicted dismal prognosis for osteosarcoma patients, and would enhance the proliferation as well as migration of osteosarcoma cells.^[17]^ Specifically, the expression of ZEB1-AS1 has been up-regulated in glioma tissues, which may indicate an increased risk of cancer progression as well as poorer OS.^[14]^ Therefore, Li et al,^[19]^ had suggested the regulatory role of lncRNA ZEB1-AS1 in non-small lung cancer (NSCLC), which might potentially serve as a new molecular marker indicating the prognosis as well as the therapeutic target for NSCLC. In this meta-analysis, high expression of ZEB1-AS1 was shown to have a strong association with poor OS and worsening prognosis including high grade tumor stage, and worsening metastasis. Therefore, this demonstrated that ZEB1-AS1 could be a potential valuable prognostic biomarker for cancers. However, the eligible studies that provided this evidence had relatively smaller sample sizes, and we could get increased power and precision if these data were obtained from different trials. Therefore, currently, in the present meta-analysis, we can only evaluate the clinical value of ZEB1-AS1 in cancer patients in China.^[28]^

In addition, the role of other factors in cancer development, metastasis, and progression should also be noted. For example, it is known that inflammation is associated with increased reactive oxygen species levels. Gaman et al,^[29]^ found that oxidative stress and decreased levels of HDL-cholesterol might play a role in diffuse large *B*-cell lymphoma (DLBCL) pathogenesis via chronic inflammation. In essential thrombocythemia (ET), chronic inflammation, and oxidative stress contribute to the genomic instability, the clonal evolution to myelofibrosis, and the leukemic transformation.^[30]^ Spiegel et al,^[31]^ observed that the abnormal lipid profile was directly related to the underlying tumor burden, particularly the presence of bone marrow involvement. Recently, Găman et al,^[32]^ also found that oxidative stress levels were higher and thrombotic events were more frequent in ET patients who had an old age at diagnosis, higher haematocrit levels or leukocytosis. Further, Iorga et al,^[33]^ reported that patients with cancer have a greater risk of both venous thromboembolism (VTE) and bleeding. A Danish retrospective study showed that patients that had VTE 1 year before the cancer diagnosis had a slightly increased risk of distant metastasis at the time of diagnosis. A total of 44% of patients who had cancer at the time of VTE had distant metastasis, with a 1-year survival rate of 12%.^[34]^ The prevalence of clinical VTE in cancer patients is 15% and is associated with poor outcomes, with a 6-fold decreased survival rate, compared with cancer patients without VTE.^[35]^

In addition, mortality outcomes seem to differ based on the ethnicity. For example, Mohamed et al,^[36]^ concluded that the overall incidence of pancreatic adenocarcinoma mortality rates among Asian–Americans was 5.740 per 100,000 person-years (95% confidence interval [CI] 5.592–5.891), and most patients were older than 60 years (77.6%) and had metastatic disease (55.8%). These findings indicate that targeting ZEB1-AS1 may affect tumor progression and metastasis; development and advanced clinical stage; and OS.

The result of this meta-analysis showed that the high expression of ZEB1-AS1 has a strong association with poor OS and worsening prognosis including high grade tumor stage, and worsening metastasis.

Therefore, ZEB1-AS1 could be viewed as a valuable prognostic biomarker for cancers in the Chinese patients. Many studies have indicated that age and sex may be associated with the incidence and progression of cancers.^[37,38,39]^ However, the result of this study did not find any significant correlation between ZEB1-AS1 expression and sex or age. May be a meta-analysis, involving a larger number of eligible studies, is needed in the future to ascertain the preceding results. In addition, more clinical studies should be conducted to evaluate potential prognostic role of ZEB1-AS1 in other types of cancer that have not been included in our meta-analysis.

Several limitations should be noted in this meta-analysis. First, it analyzed data from only 10 eligible studies focusing on a few types of cancer, and all the patients involved were from China, suggesting that the cancers reported in this study may not be representative of all cancers, and ethnic or regional differences of LncRNA expression were unclear, thus this may preclude generalizing the findings of this study to other cancers and populations. Therefore, more well-designed high quality studies should be performed in other populations of different countries to verify the role of ZEB1-AS1 among various cancer types. Second, some HRs and their corresponding 95% CIs were estimated from the survival curves, which may not be robust to perform such estimations. Finally, in future, more high quality studies should be included to avoid publication bias in similar meta-analysis.

## 5 Conclusions

The high expression of ZEB1-AS1 significantly predicated poor OS, poor metastasis and high tumor stage in this meta-analysis, demonstrating that high ZEB1-AS1 expression may serve as a biomarker of poor prognosis in the Chinese cancer patients.

## Acknowledgments

The authors thank the anonymous reviewers for helpful and meaningful comments.

## Author contributions

**Conceived and designed the analysis:** Sixiang Cheng, and Huilan Xu.

**Conceputalizaion:** Sixiang Cheng, Huilan Xu.

**Data curation:** Huilan Xu.

**Formal analysis:** Sixiang Cheng, Hairong He.

**Methodology:** Sixiang Cheng, Shengyu Guo.

**Performed the analysis:** Sixiang Cheng, Shengyu Guo.

**Software:** Shengyu Guo, Hairong He.

**Supervision:** Huilan Xu.

**Writing – original draft:** Sixiang Cheng.

**Writing – review & editing:** Sixiang Cheng, Atipatsa Chiwanda Kaminga, and Huilan Xu.

**Wrote the paper**: Sixiang Cheng and Huilan Xu.

## Supplementary Material

Supplemental Digital Content
