# Supplementary material for: Clinical value of long noncoding RNA ZEB1 anti-sense1 in cancer patients: A meta-analysis
Source: Medicine (Baltimore). 2020 Jul 31;99(31):e21307. doi: 10.1097/MD.0000000000021307 (PMC7402794; doi:10.1097/MD.0000000000021307)
Supplement: Supplemental Digital Content [file medi-99-e21307-s001.docx]

**Table S1.** Quality scores of included studies on RNA ZEB1-AS1 and OS.

|  | **Selection of Participants** | **Comparability of Groups** | **Outcomes Definition** | **Ascertainment** | **Sample Size** | **Study Design** | **Total Score** |
| --- | --- | --- | --- | --- | --- | --- | --- |
| Fu et al.(2017) | 1 | 2 | 2 | 1 | 1 | 1 | 7 |
| Gong et al.( 2017) | 1 | 2 | 1 | 1 | 1 | 1 | 7 |
| Li et al.(2017) | 1 | 2 | 2 | 1 | 1 | 1 | 8 |
| Wang et al.(2017) | 1 | 1 | 2 | 1 | 1 | 1 | 7 |
| Li et al.(2015) | 1 | 2 | 2 | 1 | 1 | 1 | 8 |
| Lin et al. (2017) | 1 | 2 | 2 | 1 | 1 | 1 | 8 |
| Lv et al.(2016) | 1 | 2 | 2 | 1 | 1 | 1 | 8 |
| Su et al.(2017) | 1 | 2 | 2 | 1 | 1 | 1 | 8 |
| Zhang et al.(2017) | 1 | 2 | 1 | 1 | 1 | 1 | 7 |
| Liu et al.(2016) | 1 | 2 | 2 | 1 | 1 | 1 | 8 |
